# Magnoflorine from *Berberis vulgaris* Roots—Impact on Hippocampal Neurons in Mice after Short-Term Exposure

**DOI:** 10.3390/ijms24087166

**Published:** 2023-04-12

**Authors:** Radosław Szalak, Małgorzata Matysek, Maryna Koval, Marcin Dziedzic, Edyta Kowalczuk-Vasilev, Marta Kruk-Slomka, Wojciech Koch, Marcin B. Arciszewski, Wirginia Kukula-Koch

**Affiliations:** 1 Department of Animal Anatomy and Histology, Faculty of Veterinary Medicine, University of Life Sciences, 12 Akademicka St., 20-950 Lublin, Poland; malgorzata.matysek@up.lublin.pl (M.M.);; 2 Department of Pharmacognosy with the Medicinal Plants Garden, Medical University of Lublin, 1, Chodźki St., 20-093 Lublin, Polandvirginia.kukula@gmail.com (W.K.-K.); 3 Department of Laboratory Diagnostics, Medical University of Lublin, 1, Chodźki St., 20-093 Lublin, Poland; 4 Institute of Animal Nutrition and Bromatology, Faculty of Animal Science and Bioeconomy, University of Life Sciences, 13 Akademicka St., 20-950 Lublin, Poland; 5 Department of Pharmacology and Pharmacodynamics, Medical University of Lublin, 20-093 Lublin, Poland; 6 Department of Food and Nutrition, Medical University of Lublin, 4a Chodźki St., 20-093 Lublin, Poland

**Keywords:** isoquinoline alkaloids, parvalbumin, hippocampus, memory, IHC, chromatography, berberidaceae, interleukins, mice

## Abstract

In search of novel potential drug candidates that could be used as treatments or prophylactics for memory impairment, an aporphine alkaloid magnoflorine (MAG) isolated from the root of *Berberis vulgaris* was proven to exhibit beneficial anti-amnestic properties. Its effects on immunoreactivity to parvalbumin in the mouse hippocampus were assessed together with a study on its safety and concentration in the brain and plasma. For this purpose, four experimental groups were created: the MAG10 group—treated with 10 mg MAG/kg b.w. *i.p.*, the MAG20 group—treated with 20 mg MAG/kg b.w. *i.p.*, the MAG50 group—treated with 50 mg MAG/kg b.w. *i.p.*, and a control group—injected with saline *i.p.* at a volume corresponding to their weight. Our results indicated that the hippocampal fields CA1–CA3 were characterized by an elevated number of parvalbumin-immunoreactive neurons (PV-IR) and nerve fibers in mice at the doses of 10 and 20 mg/kg b.w. (*i.p.*). No significant changes to the levels of IL-1β, IL-6 or TNF-α were observed for the above two doses; however, the administration of 50 mg/kg b.w. *i.p.* caused a statistically significant elevation of IL-6, IL-1beta plasma levels and an insignificant raise in the TNF-alpha value. The HPLC–MS analysis showed that the alkaloid’s content in the brain structures in the group treated with 50 mg/kg b.w. did not increase proportionally with the administered dose. The obtained results show that MAG is able to influence the immunoreactivity to PV-IR in hippocampal neurons and might act as a neuroprotective compound.

## 1. Introduction

The number of patients suffering from memory impairment incidents including dementia or developing Alzheimer’s has grown in number in recent years. Despite an increased need for efficient therapeutics that could prolong the physiological functioning of the brain, modern therapeutic strategies offer only a few drugs that are capable of slowing down the progression of the disease or work as a prophylaxis to the development of dementia [1]. First-line drugs used for the treatment of mild to moderate dementia are the inhibitors of acetyl- and butyryl-cholinesterases—the enzymes that, by decomposing acetylcholine in the synaptic cleft, alleviate the transmission of stimuli in neuronal cells. The medicines regulating the levels of the two above-mentioned enzymes can, however, bear numerous side effects or patients may develop a tolerance to the set dosage [2,3]. Lack of effective medicines that can either inhibit the cholinesterases in a more efficient way or which demonstrate another mechanism of action encouraged the researchers to search for new drug candidates of plant origin that can more efficiently cope with the memory disorders referred to as a plague of the 21st century.

The hippocampus is a key structure of the limbic system involved in properly functioning long-term memory, spatial memory, navigation, and behavioral responses. It is crucial to the entire memory process as it relates to all brain centers [4,5]. On the other hand, the hippocampus and associated structures are among the areas often damaged first in neurodegenerative disorders, of which memory impairment/dementia is the main symptom [6,7].

In neurodegenerative processes, the intracellular level of calcium ions increases dangerously. Calcium is an essential factor in the proper functioning of neurons in the central nervous system (CNS), regulating basic cellular mechanisms underlying mental processes such as learning and/or memory [8,9,10]. Calcium also plays a significant role in the pathology of CNS inflammation. Excess calcium in nerve cells is toxic, causes the degeneration of neurons, and causes the atrophy of nerve fibers, ultimately leading to the death of the cells (apoptosis) [11]. Therefore, it is important to maintain calcium homeostasis, e.g., by binding to intracellular calcium-binding proteins (CaBPs) [9]. CaBPs have the ability to buffer inflowing calcium, allowing neurons to rapidly achieve homeostasis [12]. Parvalbumin (PV), one of the CaBPs present in inhibitory GABAergic neurons, is a high-affinity Ca^2+^ binding protein that, as a buffer, plays an important role in the regulation of Ca^2+^ flow [13]. It also participates in synaptic plasticity and influences the excitability of interneurons in the hippocampus by regulating other GABAergic neurons [14].

The presented work is aimed at determining the effect of short-term exposure to magnoflorine on the intracellular level of calcium ions and the number of immunoreactive PV neurons and nerve fibers (PV-IR) in the search for novel drug candidates that could help in the treatment or prevention of memory disorders by introducing another mechanism of action from the inhibition of cholinesterase enzymes. This isoquinoline alkaloid from the aporphine subgroup is widespread in the plant kingdom among the representatives of Berberidaceae, Papaveraceae, Menispermaceae, or Magnoliaceae. It has been proven to exhibit immunomodulatory, antioxidant, anti-inflammatory, anti-anxiety, and antifungal properties [15].

Previous evidence on this alkaloid’s ability to cross the brain-blood barrier and induce central activity [16] encouraged the authors to search for good plant sources for its isolation and to deliver more detailed results on its impact on calcium homeostasis, levels of interleukins (IL-6 and IL-1beta) and a cytokine (TNF-alpha), and distribution in organ tissues (via HPLC–MS).

## 2. Results

### 2.1. Isolation of MAG from the Methanolic Extract of Berberis vulgaris Root

The analysis of the partition coefficient values (k) of the major components of *Berberis vulgaris* extracts led to the selection of a solvent system composed of chloroform: methanol: water (4:3:3 *v*/*v*) with an addition of 20 mM of hydrochloric acid and triethylamine to the upper and lower phases, respectively. In this solvent system, the calculated k values of the subsequent peaks were as follows: 873,984 for the peak at 21.04 min, 12.4 for the peak at 29.5 min, 0.25 for the peak at 32.5 min, 1.71 for the peak at 32.73 min, and 0.96 for the peak at 47.3 min (for chromatogram, see Figure S1 in the Supplementary File). Large differences between the k values of the major components of the extract promised good separation—especially that of MAG, whose peak was detected at 21.04 min and was present only in the upper phase. The behavior of the single constituents of the mixture and the affinity of all compounds except that from MAG to the lower phase led to the selection of the upper phase for the stationary one.

**Figure 1:**
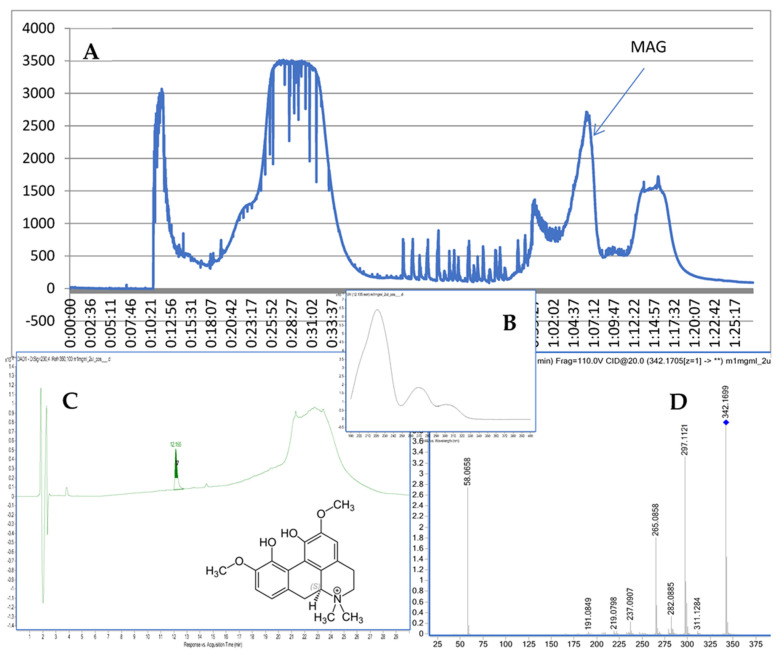
CPC chromatogram at 290 nm (**A**), the UV spectrum and structure of MAG (**B**), the purity check via HPLC (**C**), and the MS/MS spectrum of MAG (**D**).

The chromatographic separation provided 49 fractions. Their composition was first analyzed via TLC plate (see Figure S2 in the Supplementary File) and compared against the standard of MAG (Sigma Aldrich, St. Louis, MO, USA). Then, fraction 40 that showed the presence of MAG was injected on the HPLC–MS chromatograph and provided a high purity (96.2%) peak of MAG (see Figure 1).

The recorded fragmentation spectra, UV spectra and high-resolution mass measurement for the compound resembled the data that were previously published for magnoflorine [16]. The applied method provided MS/MS spectra of the compound with an *m*/*z* of 342.1699. The following major fragments were observed: 297.1121, 265.0858, and 237.0907. According to previous studies [17], they come from the detachment of NH(CH_3_)_2_ in the case of 297.1121, the following loss of an HOCH_3_ group in the case of 265.0858, and another loss of the -OC structure in the case of 237.0907. As a consequence of the fragmentation process, the ring containing nitrogen in the isoquinoline structure was opened.

### 2.2. Immunohistochemistry

**Figure 2:**
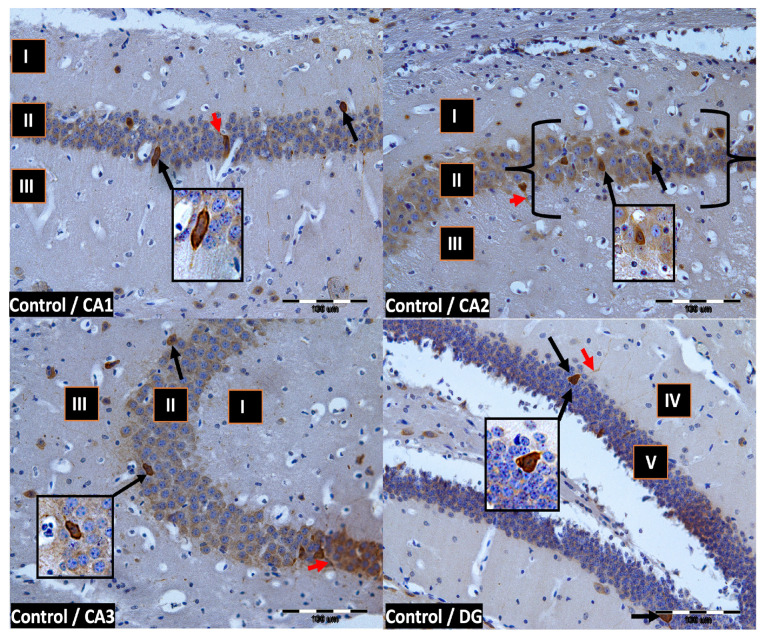
Control group. Immunoreactivity of PV-IR neurons in the fields CA1–CA3 and the DG of the mouse hippocampus was observed. I. the marginal layer, II. the pyramidal layer, III. the multiform layer, IV. the molecular layer, V. the granular layer. The arrows indicate PV-IR neurons (black arrow) and PV-IR nerve fibers (red arrow) in the hippocampus. Magnification ×20.

**Figure 3:**
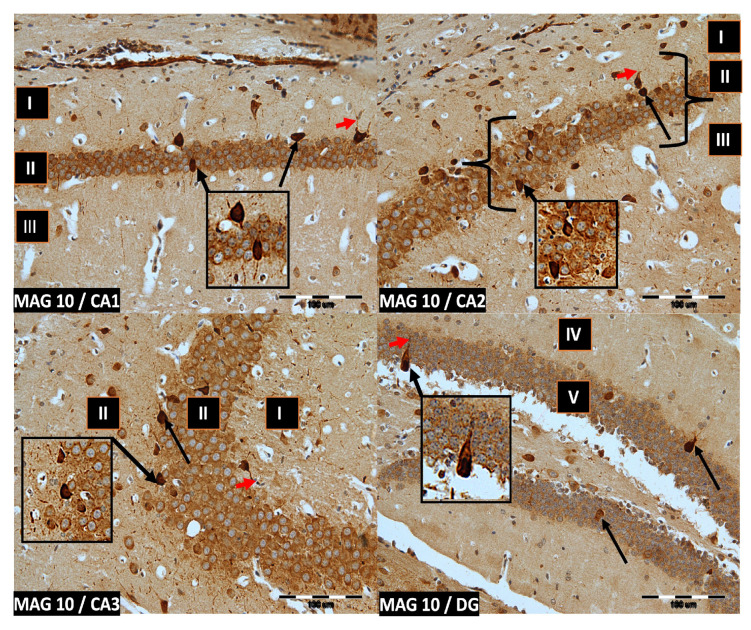
Group MAG10. Immunoreactivity of PV-IR neurons in the fields CA1–CA3 and the DG of the mouse hippocampus was observed. I. the marginal layer, II. the pyramidal layer, III. the multiform layer, IV. the molecular layer, V. the granular layer. The arrows indicate PV-IR neurons (black arrow) and PV-IR nerve fibers (red arrow) in the hippocampus. Magnification ×20.

**Figure 4:**
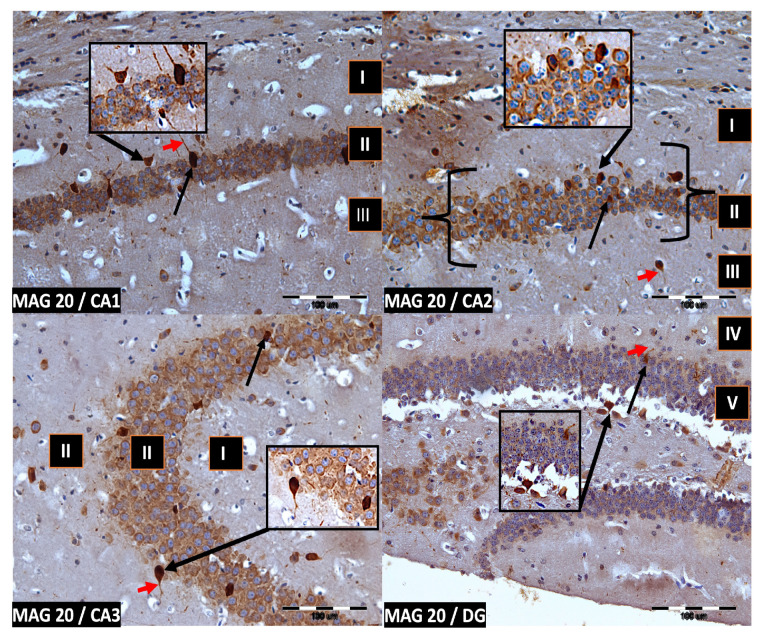
Group MAG20. Immunoreactivity of PV-IR neurons in the fields CA1–CA3 and the DG of the mouse hippocampus was observed. I. the marginal layer, II. the pyramidal layer, III. the multiform layer, IV. the molecular layer, V. the granular layer. The arrows indicate PV-IR neurons (black arrow) and PV-IR nerve fibers (red arrow) in the hippocampus. Magnification ×20.

**Figure 5:**
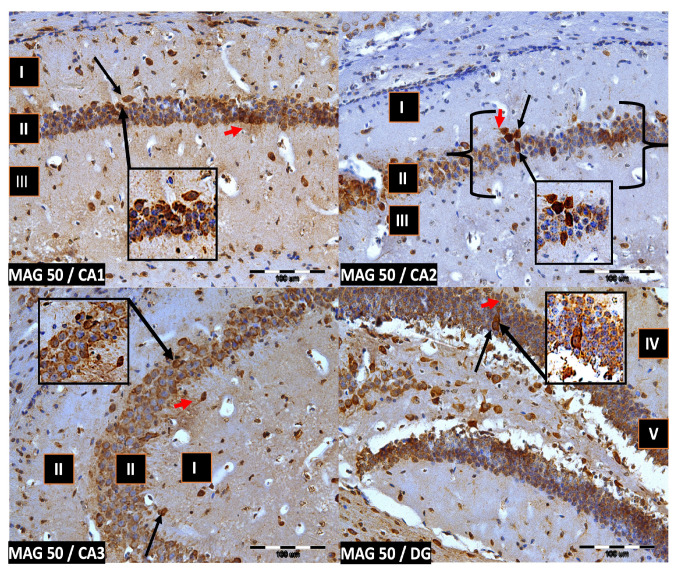
Group MAG50. Immunoreactivity of PV-IR neurons in the fields CA1–CA3 and the DG of the mouse hippocampus was observed. I. the marginal layer, II. the pyramidal layer, III. the multiform layer, IV. the molecular layer, V. the granular layer. The arrows indicate PV-IR neurons (black arrow) and PV-IR nerve fibers (red arrow) in the hippocampus. Magnification ×20.

We now describe PV immunoreactivity in the CA1–CA3 fields of the hippocampus and the DG. The hippocampus consists of the Ammon horn (CA1–CA3) and the dentate gyrus (DG). The dorsal part of the hippocampus was examined. In all study groups, multiform (oval, round, triangular, and fusiform) immunoreactivity neurons of PV (PV-IR) were observed, unevenly distributed in all layers (marginal, pyramidal, and multiform) of the mouse hippocampal fields and in the molecular and granular layer of the DG. Neurons were characterized by the presence of cytoplasmic and nuclear reactions, although the cytoplasm was more intensely stained (Figure 2, Figure 3, Figure 4 and Figure 5). The immunoreactivity to PV was also observed in the nerve fibers of both the hippocampal CA1–CA3 fields and the DG.

### 2.3. Results of the Quantitative Study

**Figure 6:**
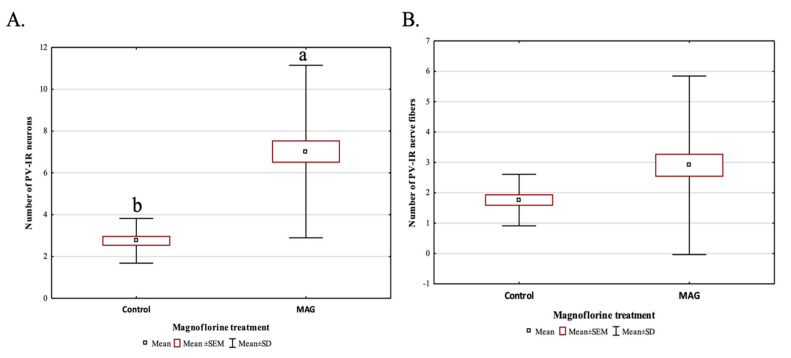
The average number of PV-IR neurons (**A**) and the average number of PV-IR fibers (**B**) in the mouse hippocampus treated with magnoflorine, irrespective of its dose. The data are expressed as means ± SEM (standard error of the mean; box) and standard deviation (whiskers); a, b—different letters indicate significant differences between the experimental groups at *p* ≤ 0.0001; n = 5.

Magnoflorine supplementation, irrespective of the dose, significantly influenced the average number of PV-IR neurons in all fields of the mouse hippocampus, as well as in the DG, compared to the control group (*p* = 0.000019; Figure 6A). On the other hand, the addition of magnoflorine, irrespective of the dose, caused an increase in the average number of PV-IR nerve fibers in the hippocampus, but no statistically significant differences were observed between the control group and the groups supplemented with magnoflorine (*p* = 0.090560; Figure 6B).

**Figure 7:**
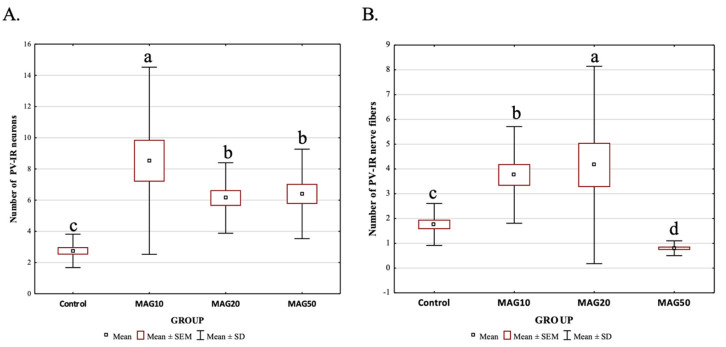
Differences in the average number of PV-IR neurons (**A**) and PV-IR nerve fibers (**B**) depending on the dose of magnoflorine (irrespective of the hippocampus field). The data are expressed as means ± SEM (standard error of the mean; box) and standard deviation (whiskers); a–d—different letters indicate significant differences between the experimental groups at *p* ≤ 0.0001; n = 5.

However, the average number of PV-IR neurons and PV-IR nerve fibers differed depending on the analyzed dose. The addition of 10 mg magnoflorine (MAG10) resulted in a significantly higher increase in the average number of PV-IR neurons in the hippocampus compared to the control and other experimental groups (MAG10 > MAG50 > MAG20 > Control). There were no statistically significant differences in the average number of PV-IR neurons between MAG20 and MAG50; however, both groups (MAG20 and MAG50) significantly differed with the control group (MAG20 = MAG50 > control group) (Figure 7A). The addition of 20 mg magnoflorine resulted in a significant increase in the average number of PV-IR nerve fibers in the hippocampus compared to the control and other experimental groups. The administration of 50 mg of magnoflorine caused a significant reduction in the average number of PV-IR nerve fibers as compared to the control group and other experimental groups, and further, additional lower doses of magnoflorine increased the average number of PV-IR nerve fibers. Statistically significant differences in the average number of PV-IR nerve fibers were also observed between the MAG10 and MAG20 groups, as well as between the MAG10 and MAG20 groups vs. the control group (Figure 7B).

**Figure 8:**
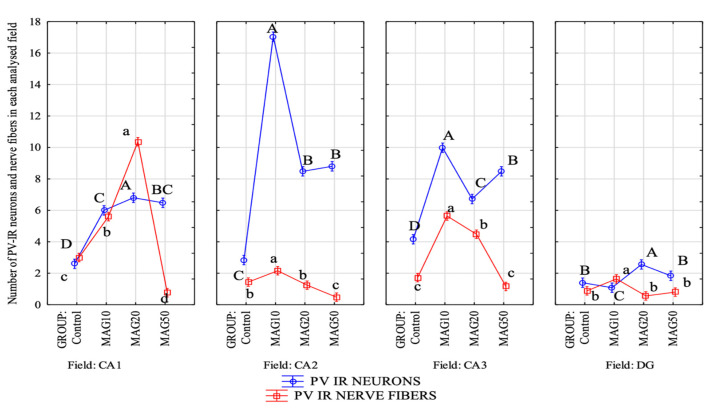
Differences in the average number of PV-IR neurons (blue line) and PV-IR nerve fibers (red line) depending on the dose of magnoflorine in different fields (CA1, CA2, and CA3) and the DG. The data are expressed as means; A, B, C, D—the different capital letters indicate significant differences at *p* ≤ 0.01 between the average number of neurons depending on the magnoflorine dose in each analyzed field; a, b, c, d—the different lower-case letters indicate significant differences at *p* ≤ 0.01 between the average number of PV-IR nerve fibers depending on the magnoflorine dose in each analyzed field; n = 5.

The magnoflorine dose also had a significant effect on both the mean number of PV-IR neurons and PV-IR nerve fibers in each analyzed hippocampal field as well as in the DG. In the CA1 field, a significant increase in the mean number of PV-IR neurons and PV-IR nerve fibers was observed in the experimental groups vs. the control group, with the greatest increase in the average number of PV-IR neurons and PV-IR nerve fibers in the MAG20 group compared to the control group, as well as compared to the MAG10 and MAG50 groups. No statistically significant differences were observed in the mean number of PV-IR neurons between the MAG10 and MAG50 groups; however, there was a significant decrease in the mean number of PV-IR nerve fibers in the MAG50 vs. the control group and vs. the MAG10 and MAG20 groups (Figure 8). In the CA2 field, a significant increase in the mean number of PV-IR neurons compared to that of the control group was observed in all experimental groups. The greatest increase in the mean number of PV-IR neurons was shown in the MAG10 group. Considering the average number of PV-IR nerve fibers in this field, a slight but significant increase was observed in the MAG10 vs. the control group and vs. the MAG20 and MAG50 groups. Moreover, a significant decrease in the mean number of PV-IR nerve fibers in the MAG50 group was demonstrated (Figure 8). In the CA3 field, as in the CA2 field, the MAG10 group showed a statistically significant increase in the mean number of PV-IR neurons and PV-IR nerve fibers vs. the control group. In the MAG50 group, a significant decrease in the average number of PV-IR nerve fibers was observed vs. the MAG10 and MAG20 groups (Figure 8). In the DG, there was a significant increase in the mean number of PV-IR neurons in the MAG20 group as compared to the control group and the other experimental groups. At the same time, a statistically significant decrease in the mean number of PV-IR neurons was observed in the MAG10 group (vs. the control group). Considering the average number of PV-IR nerve fibers, a significant increase was observed only in the MAG10 group as compared to the control group. The other experimental groups did not differ as compared to the control group (Figure 8).

The average size of the neuron was measured on three axes: vertical, horizontal, and diagonal, and after each measurement, the average size of the neuron was estimated. There were no significant differences in the average size of the PV-IR neurons in the CA1 field in the experimental groups as compared to the control group. Significant differences in the average size of the PV-IR neurons were observed between the MAG10 and MAG50 groups (MAG10 > MAG50) (Table 1). In the CA2 field, an increase in the mean size of the PV-IR neurons was observed in the MAG10 and MAG50 groups as compared to the control group, while the MAG20 group did not differ significantly from the control group and the mean size of the PV-IR neurons in this group was statistically lower than the MAG10 and MAG50 groups (Table 1). In the CA3 field, a significant increase in the mean size of the PV-IR neurons was observed in the MAG10 group (vs. the MAG20/MAG50 groups and the control group). In the remaining groups, the mean size of the PV-IR neurons did not differ to a statistically significant degree (Table 1). In the DG, a significant increase in the mean size of the PV-IR neurons was observed in the MAG10 group as compared to the control group. Additionally, a significant increase in the mean size of the neurons was observed between the MAG10 and MAG50 groups. There were no statistically significant differences between the MAG20 and MAG50 groups (Table 1).

Pearson’s correlation coefficient analysis showed that the magnoflorine addition had a significant positive effect on the average number of PV-IR neurons only in CA1 field (*p* ≤ 0.05). The average number of PV-IR nerve fibers was negatively correlated with the increasing dose of magnoflorine (Table 2). Thus, the higher the dose of magnoflorine, the average number of PV-IR nerve fibers will decrease. The analysis confirmed also that accumulation of magnoflorine in hippocampus tissue has the strongest significant impact on the CA1, CA2, and CA3 fields, but not the DG.

### 2.4. Brain and Plasma Levels of MAG via HPLC–ESI-QTOF-MS/MS

The application of HPLC–ESI-QTOF-MS/MS technology was also suitable for the determination of MAG content in the tissues and plasma of the tested mice. The chromatographic analyses that were performed on the isolated hippocampus, the remaining cortex, and the plasma showed clear data confirming the ability of MAG to cross the blood-brain barrier and localize in the brain tissue, especially in the hippocampus region. The analyzed brains and plasma came from animals treated with 10, 20, and 50 mg/kg b.w. MAG administered intraperitoneally (*i.p.*) to mice. The results of the quantitative analysis of MAG in the brain, hippocampus, and plasma are presented in Table 3.

### 2.5. Anti-Inflammatory Properties

**Figure 9:**
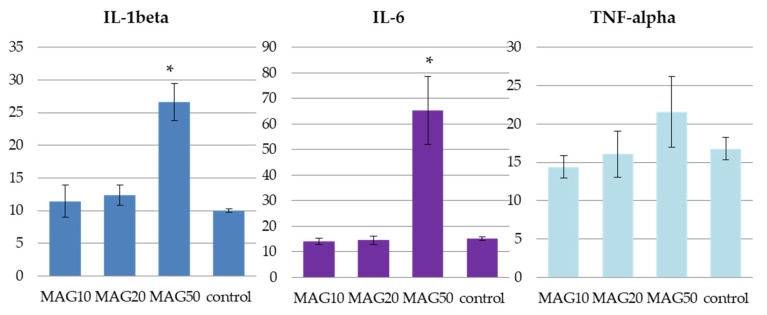
The average levels of IL-1beta, IL-6, and TNF-alpha in pg/mL of plasma of the three tested groups—MAG10, MAG20, MAG50, and a control group. The data are expressed as means ± SD (standard deviation); *—indicates significant differences at *p* ≤ 0.05 between the control group and the studied group in the *t*-test (n = 3).

The obtained values of IL-1beta, IL-6, and TNF-alpha in the studied groups of animals (MAG10, MAG20, and MAG50) and in the control group administered with saline (n = 3) showed marked differences between those that received MAG50 and the remaining samples. The administration of 50 mg/kg b.w. (*i.p.*) MAG led to a statistically significant increase in the IL-1beta and IL-6 levels. Elevation of TNF-alpha was also noted for the same group; however, the result was statistically insignificant. The two remaining groups (MAG10 and MAG20) did not exhibit major discrepancies between the treated animals and the control group (Figure 9).

## 3. Discussion

Centrifugal partition chromatography belongs to hydrostatic counter-current chromatography—a technique that uses a biphasic solvent system to fractionate mixtures based on the affinity of individual components to either the lower or upper phase [18]. This technique has been proven to be particularly efficient in the purification of alkaloids. These nitrogen-containing molecules tend to adsorb on classical beds like silica gel that fills the majority of chromatographic columns. This occurrence results in tailing during the purification process and a difficult isolation process. Lack of solid support and the ability to introduce modifiers—such as acids and bases—to the purification system supports the recovery of alkaloids from rich plant matrices using this technique. Previously, the scientific literature delivered several examples of alkaloid purification. Nakonieczna et al. [19] succeeded in the purification of isoquinoline alkaloids from *Coptis chinensis*; benzylisoquinoline and aporphine alkaloids were recovered from *Spirauna glycycarpa* [20] using a polar solvent system composed of n-butanol-methanol-water (9:1:10, *v*/*v*); *O*-tigloylcyclovirobuxeine-B was obtained via CPC fractionation of *Buxus sempervirens* extract with a hexane:EtOAc:MeOH:H2O (7:3:7:3) (*v/v/v/v*) solvent system [21], whereas aconitine was purified from *Aconitum karacolicum* by Tarbe et al. [22]. In the case of this study, a composition of the following solvents: chloroform:methanol:water (4:3:3 *v*/*v*), provided high purity MAG directly from the crude methanolic root extract of *Berberis vulgaris*. It is also important to add that the described technique offers the possibility of upscaling the methodology and is suitable for high throughput purification of single compounds from complex matrices, such as plant extracts [23].

Parvalbumin is a protein with a relatively high affinity for Ca^2+^ ions; therefore, the role of PV is to buffer Ca^2+^, transport these ions, and protect neurons from excessive calcium accumulation [24]. It is assumed that each modification of CaBPs as a consequence of, for example, neurodegenerative diseases, brain aging, or diabetes, disturbs calcium homeostasis in the cell, leading to the pathological accumulation of mitochondrial Ca^2+^ levels, which, in turn generates neuronal susceptibility to apoptosis. Wöhr et al. [25] suggest that a lack of PV immunoreaction is sufficient to cause behavioral abnormalities. Therefore, it is reasonable to conclude that neurons containing CaBPs are probably more resistant to damage due to their ability to buffer calcium.

In the available literature, there is no shortage of information about the differentiated immunoreactivity of PV during various neurodegenerative disorders. Brady and Mufson [26] observed that there was an approximately 60% decrease in the number of PV interneurons in the hippocampus of people with Alzheimer’s disease (AD), although not all hippocampal fields were equally affected. They suggested that PV interneurons from different fields of the hippocampus are selectively susceptible to AD. The population of hippocampal interneurons is also reduced in the aging brain in both rats and humans [27]. Likewise, Ryan and Geckle [28] observed changes in hippocampal interneurons in animal models of diabetes.

In the present work, we evaluated the immunoreactivity of PV to investigate the effect of magnoflorine on the immunoreactive neurons of the hippocampus as a specific memory and reasoning center. In previous work [16], we found that MAG improves cognitive functions in the PA test for short- and long-term memory. The assay was planned to collect mice organs directly after decapitation. This procedure follows previously published results which indicate that the biological half-life concentration of a MAG injection in animal plasma is around 4 h [29]. That is why the authors expected maximal concentration to occur around 1–2 h after the administration of the alkaloid.

We have now found that MAG supplementation, regardless of the dose, significantly affected the mean number of PV-IR neurons compared to the control group. However, there were no statistically significant differences in the average number of PV-IR nerve fibers. Considering the dose of magnoflorine, a significant increase in the average number of PV-IR neurons and PV-IR nerve fibers was observed in the MAG10 and MAG20 groups. Interestingly, at a dose of 50 mg of magnoflorine, there was a significant decrease in the mean number of PV-IR nerve fibers compared to both the control group and the other experimental groups.

The addition magnoflorine also significantly affected the average number of PV-IR neurons and PV-IR fibers depending on the field of the hippocampus. In the CA1 field, we noted a significant increase in the mean number of PV-IR neurons in all experimental groups (vs. the control group), although it was the largest in the MAG20 group. Similarly, in the CA2 and CA3 fields, where we observed a significant increase in the average number of PV-IR neurons in all the experimental groups, the difference was that the largest increase was seen in the MAG10 group. Considering the average number of PV-IR nerve fibers in all hippocampal fields, a significant increase was observed in MAG10, but, interestingly, we noted a significant decrease in the average number of PV-IR nerve fibers at the dose of magnoflorine 50 mg (vs. the control group and other experimental groups). Moreover, MAG administration caused changes in the average size of the PV-IR neurons in the hippocampal fields and DG.

A similar correlation between the increase in the average number of PV-IR neurons in the experimental groups and the control group was observed in our previous work describing the effect of another isoquinoline alkaloid, berberine, on PV neurons in mouse hippocampal fields [30].

The above-mentioned evident effects on the stimulation of the number and size of neurons by MAG suggests its beneficial role in the brain structures. Importantly, the impact of MAG on calcium homeostasis, through its influence on PV-IR neurons and PV-IR nerve fibers, may have a beneficial impact on patients suffering from diabetes or dementia. Even minor changes and/or disruptions in Ca^2+^ transport systems can seriously affect intracellular calcium ion levels, causing nerve cell dysfunction and ultimately leading to apoptosis.

The aforementioned observed decline in the number of neurons in the MAG50 group of animals may be due to the side effects of the highest dose. A previous publication of the authors shows a disturbance in the locomotor activity of mice [16] under the influence of the same 50 mg/kg dose, whereas the herein described results of the anti-inflammatory assays show evidence of an increase in the IL-6 and IL-1 beta plasma levels together with a statistically insignificant increase in the TNF-alpha level that all could result from intoxication. The determination of MAG content in the brain, hippocampus, and plasma shows the highest brain/hippocampus content of MAG in the MAG10 group. Interestingly, the MAG50 group of animals did not exhibit any increase in MAG concentration that could be proportional to its elevated dose. The results for the MAG50 group were obtained during the initial dose administration studies. Due to insufficient information on an effective dose of MAG that could be used in the study, the authors selected three doses: 10, 20, and 50 mg/kg *i.p.* to be administered to animals. The final one was found to exhibit undesired effects in the study.

The delivered results on the impact of MAG on the serum levels of cytokines and tumor necrosis factor alpha, together with the quantitative analysis of its content in the brain tissue and plasma, resemble former studies on different compounds from the same group of metabolites. In studies by other authors, different isoquinoline alkaloids were found to exhibit anti-inflammatory or protective roles in animal models with induced inflammatory conditions. Based on the former evidence, the unchanged serum levels of interleukins and TNF-alpha in the MAG10 and MAG20 groups are not surprising. Previously, palmatine was proved to decrease inflammatory biomarkers like TNF-alpha and IL-6 in the study by Li and his co-investigators at the dose of 20 mg/kg b.w. intragastrically (*i.g.*) [31]. Also, berberine exhibited a similar tendency and reduced the IL-1beta, IL-6, and TNF-alpha levels in the study on mice with induced ulcerative colitis, at a dose of 100 mg/kg b.w. *i.g*. [32]. This comparison with previously published literature explains the bioactivity of magnoflorine.

The pathological changes of AD are complex and diverse. However, it has been known that inflammation and oxidative stress play a key role in the pathogenesis of this illness. It has been reported that intense oxidative stress in the brain is one of the main causal factors connected with AD-related memory impairment through two critical changes in the brain. First, a decrease in a neurotransmitter essential for memory and learning functions, acetylcholine, and second, a decrease in the level of natural antioxidants in the brain by activating microglia, a source of reactive oxygen species (ROS), has been described. CNS is very susceptible to processes related to oxidative stress. Consequently, oxidative stress on nervous tissue may seriously damage the brain via several interacting mechanisms, including the release of excitatory amino acids or increase in intracellular free Ca^2+^. Moreover, ROS are highly neurotoxic and thereby induce oxidative damage in connection with many neurodegenerative disorders, for example, AD. Additionally, free radicals created by upregulated production of pro-inflammatory factors, such as cytokines and chemokines, could trigger neuroinflammation. In particular, TNF-α and IL-1beta can provoke chronic inflammation that causes the loss of synapses, neuronal death, and consequently, memory dysfunction characteristic of AD [33].

Therefore, based on the fact that oxidative stress and inflammation can result in AD-related dysfunctions, the drugs that are able to inhibit oxidative and inflammatory processes could be very promising for the treatment of AD.

MAG is one of the most active plant compounds. The pharmacological effects of MAG have been extensively reported. Literature data have described a variety of pharmacological effects of this plant compound, including antidiabetic, antiallergic, anticancer, antifungal, or antiviral activities, as well as anti-inflammatory and antioxidant properties [34]. Moreover, considering the obtained results, it is suggested that MAG penetrates the blood-brain barrier. The previous findings of Li and his co-investigators [35] underline the ability of MAG to exhibit central action—either alone or in conjunction with a lipophilicity-lowering phospholipid complex. MAG crosses the blood-barrier, affecting the regulation of the central nervous system (CNS). In the context of our manuscript, the widely neuropsychopharmacological effect of MAG seems to be the most important in the context of the aforementioned potential therapeutic strategies for many CNS diseases, especially AD.

The antioxidant and anti-inflammatory effect of MAG has been evaluated previously [36]. This herbal compound is able to inhibit the inflammatory production of nitric oxide (NO) and protect murine macrophage cells (RAW 264.7) from lipopolysaccharide-induced apoptosis. Moreover, it has been found that MAG caused a decrease in the expression of typical pro-inflammatory cytokines (TNF-α, IL-1β, and IL-6) in a dose-dependent manner.

Our present study also confirmed that MAG exhibits a strong anti-inflammatory potential that is in agreement with that found by other authors [37]. Additionally, our previous study, along with reported data, described the cognition-enhancing and anti-amnestic effects of MAG [16]. Moreover, our other study revealed the anticholinesterase activity of MAG in an in vitro thin-layer chromatography (TLC)-based bioautographic study of *Argemone mexicana* L. extracts that could have a positive influence on the acetylcholine level in the brain [38].

Analyzing the above-mentioned properties of MAG and the results presented in the following paper, we can suggest that MAG, through its complex mechanism of action connected with most of the AD pathogenesis processes, may be a good potential therapeutic candidate for AD. However, further studies are needed.

## 4. Materials and Methods

### 4.1. Plant Material and Extraction

Ground root of *Berberis vulgaris* distributed by the Proherbis company (Proherbis Jaroslaw Wolanski, Debowiec, Poland) was purchased in a local herbal shop in Lublin, Poland. The extract was obtained in an accelerated solvent extractor (ASE 100, Dionex, Sunnyvale, CA, USA) using a preparative cell with 30 g of plant material in the following conditions: extracting solvent: methanol, extraction temperature: 80 °C, static cycle length: 5 min, number of static cycles: 3, operation pressure: 105–110 bar, purge time: 30 s, and flush volume: 30%. Later, the obtained extract was evaporated to dryness using a rotary evaporator at a temperature of 45 °C. The dried residue was weighted and used for the recovery of MAG.

### 4.2. Centrifugal Partition Chromatography-Based Separation of Magnoflorine

The selection of the biphasic solvent system suitable for the isolation of MAG from the *Berberis vulgaris* root methanolic extract was performed first. Several biphasic solvent systems at a volume of 5 mL were prepared in a tube. Then, 20 mg of extract was mixed in every system. Later, equal volumes of the upper and lower phases were transferred separately to HPLC vials and injected on a chromatographic column. The chromatographic analysis of the upper and lower phases’ composition—separately, provided evidence for the calculation of *k*—partition coefficient. This factor showed the distribution of a given compound between the upper and lower phase, as it comes from the division of peak areas of compounds present in the lower phase by the corresponding peak areas in the upper phase. The obtained values helped us to understand the potential efficiency of separation in the given conditions.

The chromatograms were recorded using a Shimadzu chromatograph (Kyoto, Japan) that was composed of a degasser (DGU-20A), a quaternary pump (LC-20CE), a column thermostat (CTO-10AS VP,) and a PDA detector (SPD-M 20A). For the purpose of separation, an RP-18 chromatographic column Kinetex (Phenomenex, Torrance, CA, USA) with the dimensions of 250 mm × 4.6 mm and a grain of 5 µm was used. Run time was set at 60 min, the flow rate at 1 mL/min, and the thermostat temperature at 20 °C. A gradient of 1% acetic acid in acetonitrile (solvent B) in 1% acetic acid was used: 0 min: 10% of B, 20 min: 40% of B, 40–45 min: 95% of B, and 46 min: 1% of B.

The selected biphasic solvent system composed of chloroform:methanol:water (4:3:3 *v/v*) with an addition of 20 mM of hydrochloric acid and triethylamine to the upper and lower phases, respectively, (see Figure S1 in the Supplementary File) was used for the chromatographic fractionation of the extract on the CPC chromatograph (SCPC-250-L, Armen Instruments, Saint Ave, France). The instrument was equipped with a 250 mL stainless steel column, a UV detector, and a fraction collector. The upper phase was selected as the stationary phase, whereas the lower one was selected as mobile, and the isolation was performed in the pH-zone refining mode of operation that makes use of the ability of alkaloids to be present in the acid and base forms.

First, 500 mg of extract was dissolved in 5 mL of 70:30 *v/v* mixture of upper to lower phases with the addition of trietylamine, but with no hydrochloric acid. Then, the column was filled with the stationary acidified phase at a flow rate of 20 mL/min with a rotation speed of 500 rpm for 15 min. After completion of this step, the sample was injected together with the basified mobile phase at a rotation speed of 1300 rpm and a flow rate of 6 mL/min for 50 min and 8 mL/min for the remaining 36 min. The total separation time lasted for 86 min and was conducted in the elution mode for the first 50 min and in the extrusion mode for the last 36 min. The composition of the eluate was monitored at two wavelengths: 254 and 290 nm. A total of 12 milliliter-volumed fractions were collected throughout the analysis and analyzed for their composition via TLC, HPLC, and HPLC–MS.

The HPLC–MS analysis provided information about the purity and mass spectrum of magnoflorine. The experiments were recorded in the Agilent Mass Hunter Data Acquisition program version 10.1 (Agilent Technologies, Santa Clara, CA, USA) using an instrument composed of a degasser, binary pump for solvent (G1329B), isocratic pump for reference ions (G1310B), an autosampler, UV detector, and Q-TOF detector with an electro-spray ionization source (G6530B). The operational conditions for the HPLC–MS were as follows: gas temperature: 275 °C, sheath gas temperature: 325 °C, gas flow and sheath gas flow: 12 L/min, nebulizer pressure: 35 psig, capillary voltage: 3000 V, nozzle voltage: 1000 V, fragmentor voltage: 110 V, skimmer voltage: 65 V, solvent flow rate: 0.2 mL/min, reference ions flow rate: 0.050 mL/min, thermostat temperature: 20 °C, UV wavelengths: 210, 254, 290, and 365 nm; and gradient of solvent B (acetonitrile with 0.1% formic acid) in 0.1% formic acid: 0 min—10% of B, 10 min—20% of B, 15 min—40% of B, 17–18 min—95% of B, and 19–30 min—10% of B. The MS/MS spectra were obtained from two the most intensive spectra at two collision energies: 10 and 20 V. Agilent Mass Hunter Qualitative Navigator version B.10.00 was used to handle the recorded data and the Zorbax Eclipse Plus RP-18 chromatographic column (150 mm × 2.1 mm, 3.5 μm) by Agilent Technologies (Santa Clara, CA, USA) was used to facilitate the separation of metabolites.

### 4.3. Animals

Naïve male Swiss mice weighing 20–30 g were obtained from the Farm of Laboratory Animals (Warszawa, Poland). The animals, in groups (n = 8–9), were maintained under standard laboratory conditions (12 h light/dark cycle at a room temperature of 21 ± 1 °C). They had free access to water and laboratory chow (Agropol, Motycz, Poland) inside their cages and were left to adapt to the laboratory conditions for a period of one week. Four groups of animals were tested: MAG10—injected with 10 mg/kg b.w. *i.p.* magnoflorine, MAG 20—injected with 20 mg/kg b.w. *i.p.* magnoflorine, MAG50—injected with 50 mg/kg b.w. *i.p.* magnoflorine, and control—injected with saline at a volume corresponding to their weight, *i.p.* All injections were made one time to mimic an acute exposure to the tested substance or saline. Thirty minutes after the administration, the animals were directed to a passive avoidance test and after testing (1–2 h after injection) they were sacrificed (beheaded) using a guillotine. The protocol was described by the authors in a previous publication [16]. All experiments were performed according to the National Institute of Health Guidelines for the Care and Use of Laboratory Animals and to the European Community Council Directive for the Care and Use of Laboratory animals of 22 September 2010 (2010/63/EU) and were approved by the local ethics committee.

### 4.4. Drugs

Magnoflorine isolated from the methanolic root extract of *Berberis vulgaris* was transferred to a mortar and dissolved in saline on the same day prior to intraperitoneal administration (*i.p.*). Fresh solutions of 10, 20, and 50 mg/kg b.w. doses of magnoflorine were used, whereas the control group was given a corresponding volume of saline.

### 4.5. Immunohistochemistry and Antibodies

Following euthanasia (decapitation) that was performed 1 hour after the administration of the solution, the mice’s brains were stored in 10% buffered formalin (pH = 7) for 12 h at 4 °C, hydrated in decreasing concentrations of ethyl alcohol, and embedded in paraffin blocks in accordance with the previously described method [39]. Finally, the paraffin blocks were cut into 5 μm thick sections with a microtome (Microm HM 360, Microm, Walldorf, Germany). Sections were placed on adhesive glass slides (Superfrost Plus, Thermo Scientific, Braunschweig, Germany). The slides were immunohistochemically stained (peroxidase-antiperoxidase method) according to the following protocol. First, in order to remove paraffin, the sections were washed in xylene (3 × 15 min). Then, the slides were rehydrated via sequential incubation with a graded series of ethyl alcohol, and finally they were washed in distilled water. The slides were put in a container containing citrate buffer (pH = 6) and heated to 97 °C (3 × 7 min) in a microwave oven (800 W) in an attempt to retrieve antigens. Selected sections were outlined with a hydrophobic marker (ImmEdge™ Hydrophobic Barrier Pen, Vector Laboratories, Burlingame, CA, USA). In order to block the endogenous peroxidase activity, the sections were chilled and washed in 3% hydrogen peroxidase (20 min). The slides were then flushed twice with PBS (pH = 7.4) (15 min each time) and incubated in 2.5% normal goat serum (ImPRESS^TM^, MP-7451, Vector Labs, Burlingame, CA, USA) at RT for 20 min. Next, the sections were incubated for 24 h at 4 °C with primary monoclonal rabbit antibodies raised against PV (1:2000; SWANT, PV25, Fribourg, Switzerland). The next day, the slides were washed in a washing buffer (2 × 15 min) and covered with anti-rabbit Ig (ImPRESS^TM^, MP-7451 Vector Labs, Burlingame, CA, USA) for 1 h. For the visualization of primary antisera, 3,3′-diaminobenzidine (ImmPACT^TM^ DAB, SK-4105, Vector Labs, Burlingame, CA, USA) chromogen was used. Moreover, counterstaining (for 20 min) was performed with Mayer’s hematoxylin. After washing in distilled water, the sections were dehydrated in increasing concentrations of ethyl alcohol, cleared in xylene, mounted in DPX (Sigma-Aldrich, St. Louis, MO, USA), and cover-slipped. Negative control was used to test the specificity of the antibodies. Sections considered to be negative controls were not exposed to primary antibodies (omitted or replaced with non-immunoreactive sera). No positive immunoreaction was detected in any of the control procedures.

The slides were viewed under a light microscope (Axiolab, Zeiss, Jena, Germany). Images were acquired using a digital camera (C11440-36U, Hamamatsu Photonics, Shizuoka, Japan) connected to a standard PC with Cell^M 2.3 image analysis software ver. 13.1 (Olympus cellSens Standard). Images were taken under a 20× objective at a resolution of 1024 × 1024 pixels. From each animal, approx. 25–30 of immunostained sections for PV were examined.

The average numbers of PV-IR neurons were assessed by analyzing and counting no less than one hundred neurons in every field (CA1–CA3) and the DG of the hippocampus of the control and experimental groups. In addition, measurements of these PV-IR neurons in all hippocampal fields and the DG were made in three cross-sections: vertical, horizontal, and diagonal, calculating the average size of the neurons. Morphometric analysis of the PV-IR neurons was performed using Cell^M 2.3 software (Olympus cellSens Standard).

The average number of PV-IR nerve fibers were also counted among no less than one hundred neurons with and without PV-IR reactions in every field (CA1–CA3) and the DG of the hippocampus of the control and experimental groups using Cell^M 2.3 software (Olympus cellSens Standard). At least two independent observers were involved in quantification analyses and the results obtained by them were averaged.

### 4.6. The HPLC–ESI-QTOF-MS Quantitative Analysis of Mice Brains and Plasma

The compositional studies of MAG in the brain tissues and plasma (n = 3) were conducted using a similar methodology as described above (HPLC–MS analysis in Section 4.2). A total of 50 microliters of plasma were mixed with the same volume of cold acetonitrile and vortexed for 10 min. Then, a portion of 50 µL of distilled water was added, mixed again, centrifuged for 20 min at 12,000 rpm, and filtered through nylon syringe filters with a 0.2 µm pore diameter to an HPLC vial. The brain without the hippocampus and hippocampus alone of every tested animal were separately homogenized on ice (PRO 200 Bio-Gen Series tissue homogenizer, Pro Scientific Inc., Oxford, UK) with acetonitrile (100 µL of acetonitrile in the case of brains and 50 µL for the hippocampus). Then, the following 100 μL (per brain) or 50 µL (per hippocampus) of water was added, mixed, and the samples were centrifuged similar to the plasma. The supernatant was collected in HPLC vials through a similar 0.2 µm pore size syringe filter. The samples were kept at −80 °C prior to analysis. The quantification of MAG in the analyzed biological material was based on the calibration curve of a >95% purity standard (Sigma Aldrich, St. Louis, MO, USA) with a regression value of R^2^ = 0.9934 within the tested range of concentrations: 0.0005–0.1 mg/mL.

### 4.7. Determination of Anti-Inflammatory Properties

The levels of IL-6, IL-1beta, and TNF-alpha were determined from 50 µL of plasma samples obtained from three animals from each tested group: MAG10, MAG20, MAG50 and the control using commercially available enzyme-linked immunosorbent assays kits using the protocol recommended by the producer—Diaclone Immunology Products & Service (Besançon, France). In the study, murine TNF alpha, mouse IL-1beta, and murine IL-6 ELISA kits were used.

### 4.8. Statistical Analysis

All the data were analyzed with Statistica software ver. 13.1 (StatSoft, Kraków, Poland). Normality was assessed using the Kolmogorov–Smirnov test, and Levene’s homogeneity of variance test was applied to examine the equality of variances. One-way ANOVA was performed to assess the impact of magnoflorine treatment (irrespective of its dose) as compared to the non-treated control group. Two-way ANOVA was used to assess the effect of the magnoflorine dose (0 mg, 10 mg, 20 mg, or 50 mg), depending on the analyzed field of the hippocampus (CA1, CA2, or CA3) and the DG, on the mean number and mean size of the PV-IR neurons, as well as on the mean number of PV-IR nerve fibers. *Post-hoc* Tukey’s analysis was performed to estimate the significant differences between the groups at a significance level of *p* ≤ 0.05 and *p* ≤ 0.01. Additionally, to assess the strength of the relationship between the magnoflorine treatment and the average number of PV-IR neurons and nerve fibers, the Pearson correlation coefficients were estimated at the significance level of *p* ≤ 0.05. Student’s *t*-test was performed to evaluate the statistically significant differences between the parameters of the plasma in every tested group of animals with the control group with a significance level of *p* ≤ 0.05.

## 5. Conclusions

In conclusion, magnoflorine, the aporphine alkaloid of interest, could be successfully isolated from the plant matrix (the root of *Berberis vulgaris*) by means of centrifugal partition chromatography. The elaborated composition of solvents provided magnoflorine in the eluate after one hour of fractionation at a quantity suitable for the conduction of in vivo studies on mice.

Based on the analysis of the Pearson’s correlation coefficient, we can conclude that the administration of magnoflorine had a significant positive effect on the mean number of PV-IR neurons only in the CA1 field. This field is characterized by a high sensitivity of nerve cells to damage compared to the hippocampal CA2 and CA3 fields. However, the decrease in the mean number of PV-IR nerve fibers at a higher dose of MAG (50 mg) may suggest a negative effect of this alkaloid on neuronal Ca^2+^ metabolism at the highest dose, which may possibly lead to neurotransmission disorders. This mechanism may be due to the intoxication induced by the highest tested dose of the alkaloid. The tested plasma levels of IL-6 and IL-1beta were elevated and significantly differed from the control group and groups of animals administered with two lower doses: 10 and 20 mg/kg b.w.. Also, the level of TNF-alpha was elevated for the MAG50 group, but the difference with the control group was insignificant. The HPLC–MS analysis of brain homogenates showed only a slight increase in the brain tissue concentration of magnoflorine in the MAG50 group in comparison to the other groups.

Undoubtedly, further research is needed on the possible neuroprotective role of MAG and other isoquinoline alkaloids.

## Figures and Tables

**Table 1:** The average sizes of PV-IR neurons depending on the the magnoflorine doses (10, 20 and 50 mg) in each investigated field (CA1, CA2, and CA3) and the DG. The data are expressed as means ± SD (standard deviation); a–c—the different letters indicate significant differences at *p* ≤ 0.01 between the average neuron size depending on the magnoflorine dose in each field; n = 5.

Field	Group				SEM	Impact of		
	Control	MAG10	MAG20	MAG50		MAG	Field	MAG × Field
CA1	12.78ab ± 2.51	13.81a ± 2.90	12.72ab ± 2.38	11.09b ± 1.38	0.647	0.00001	0.015	0.014
CA2	11.47 ± 1.85	12.69 ± 1.69	11.38 ± 0.83	12.53 ± 1.82	0.469			
CA3	11.39b ± 1.50	14.28a ± 1.63	12.31b ± 1.93	12.33b ± 1.29	0.498			
DG	12.59bc ± 1.48	14.45a ± 1.95	12.01bc ± 2.06	13.29b ± 1.93	0.502			

**Table 2:** Pearson’s correlation coefficients (r) between the dose of magnoflorine and its concentration in the mouse hippocampus and its impact on PV-IR neurons and the number of PV-IR nerve fibers in different fields (CA1, CA2, and CA3) and the DG. Values of correlation coefficients in bold are statistically significant (*p* ≤ 0.05).

Pearson’s Correlation Coefficient	MAG Dose	MAG Concentration
MAG concentration	**0.47**	
No. of PV-IR neurons		
Total	0.17	**0.43**
CA1 field	**0.64**	**0.95**
CA2 field	0.10	**0.71**
CA3 field	0.44	**0.75**
DG	0.37	0.40
No. of PV-IR nerve fibers		
Total	**−0.25**	**0.29**
CA1 field	−0.35	**0.52**
CA2 field	**−0.79**	**0.78**
CA3 field	−0.40	**0.60**
DG	−0.24	0.05

**Table 3:** The content of MAG in the tested biological material: hippocampus, brain and plasma [mg/mL].

Biological Material	Group		
	MAG10	MAG20	MAG50
Brain	0.0105 ± 0.001	0.00559 ± 0.0001	0.00760 ± 0.0007
Hippocampus	0.00147 ± 0.00005	0.00128 ± 0.00004	0.00171 ± 0.0001
Plasma	0.00212 ± 0.0004	0.00139 ± 0.0002	0.00134 ± 0.00007

## Data Availability

The additional information is located in the Supplementary File.

## Supplementary Materials

The following supporting information can be downloaded at: https://www.mdpi.com/article/10.3390/ijms24087166/s1.
