# Supplementary material for: Magnoflorine from Berberis vulgaris Roots—Impact on Hippocampal Neurons in Mice after Short-Term Exposure
Source: Int J Mol Sci. 2023 Apr 12;24(8):7166. doi: 10.3390/ijms24087166 (PMC10138352; doi:10.3390/ijms24087166)

Supplementary File

Figure S1. Chromatograms of the upper and lower phases of the selected solvent system recorded at 254 nm with magnoflorine at 21 min.

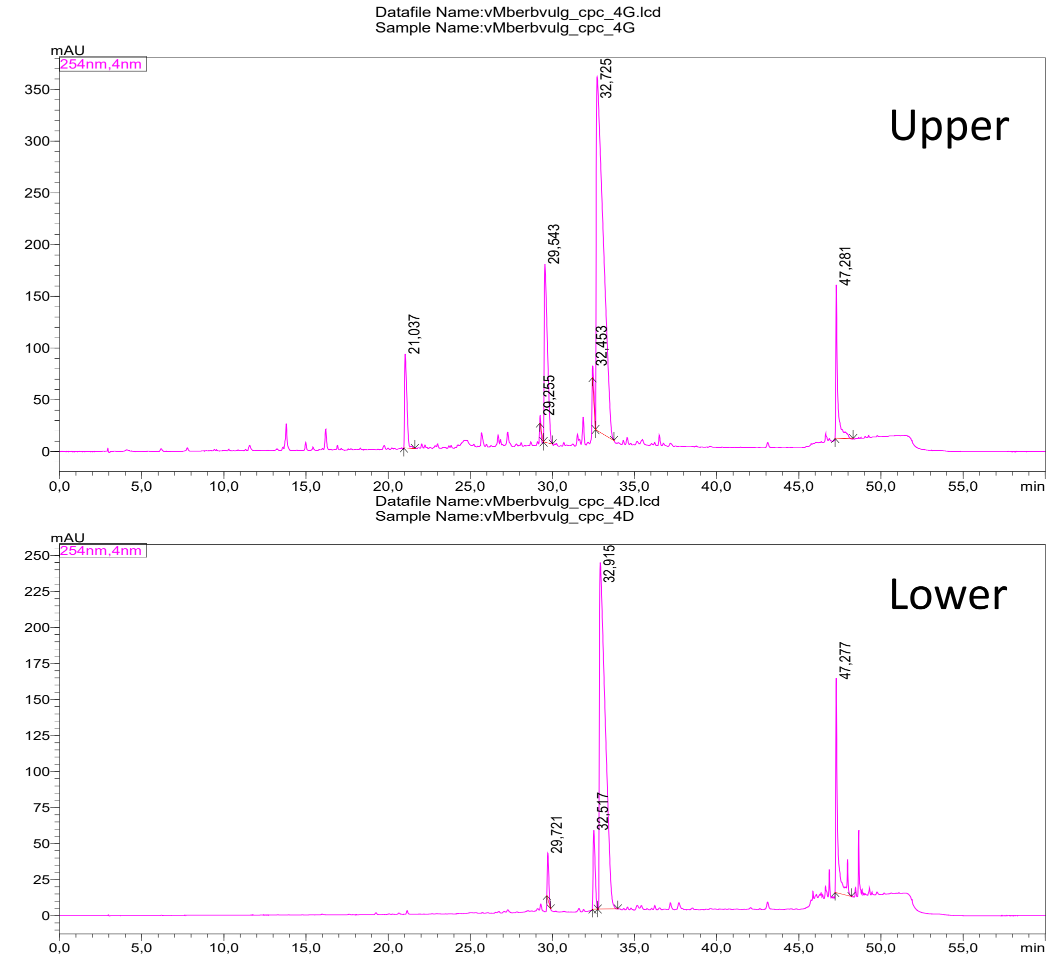

Figure S2. TLC chromatogram at 254 nm of a normal phase plate with fraction 40 rich in MAG and MAG standard on the right

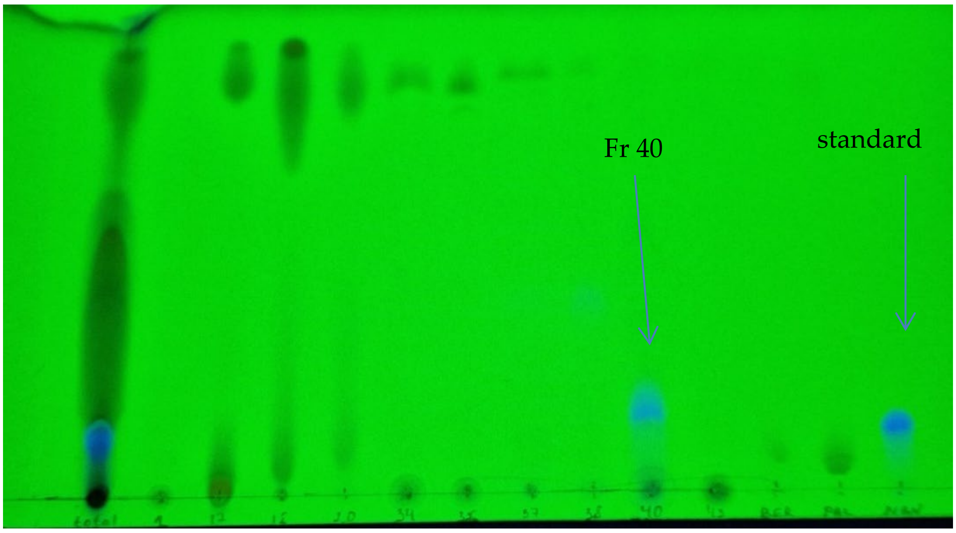

Supplement: Supplementary file 1 [file ijms-24-07166-s001.zip › ijms-2333699-supplementary.pdf]
